# Protocol for the Exercise, Cancer and Cognition – The ECCO-Study: A Randomized Controlled Trial of Simultaneous Exercise During Neo-/Adjuvant Chemotherapy in Breast Cancer Patients and Its Effects on Neurocognition

**DOI:** 10.3389/fneur.2022.777808

**Published:** 2022-03-25

**Authors:** David Kiesl, Marina Kuzdas-Sallaberger, David Fuchs, Silvana Brunner, Romana Kommenda, Clemens Tischler, Herwig Hornich, Kaveh Akbari, Jörg Kellermair, Hermann Blessberger, Helmuth Ocenasek, Peter Hofmann, Philipp Zimmer, Milan R. Vosko

**Affiliations:** ^1^Department for Internal Medicine III, Kepler University Hospital, Linz, Austria; ^2^Cardiomed, Cardiological Rehabilitation, Linz, Austria; ^3^Department for Palliative Care, Ordensklinikum Linz, Sisters of Mercy Hospital, Linz, Austria; ^4^Department for Clinical Psychology, Kepler University Hospital, Linz, Austria; ^5^Central Radiology Institute, Kepler University Hospital, Johannes Kepler University, Linz, Austria; ^6^Department of Cardiology, Medical Faculty of the Johannes Kepler University, Kepler University Hospital, Linz, Austria; ^7^Institute of Human Movement Science, Sport & Health, Exercise Physiology, Training & Training Therapy Research Group, University of Graz, Graz, Austria; ^8^Divison of Performance and Health (Sports Medicine), Institute for Sport and Sport Science, TU Dortmund University, Dortmund, Germany; ^9^Department of Neurology, Kepler University Hospital, Linz, Austria

**Keywords:** exercise, breast cancer, cognition, physical activity, cancer related cognitive impairment (CRCI)

## Abstract

**Introduction:**

Epidemiological studies show that increased physical activity is linked to a lower risk of breast cancer and mortality. As a result, physical activity can significantly improve patients' quality of life (QOL) both during and after therapy.

Many breast cancer patients demonstrate a decrease in cognitive capacity, referred to as the symptom-complex cancer related cognitive impairment (CRCI). Most frequently reported impairments are mild to moderate deficits in processing speed, attention, memory, and executive functions. Cognitive symptoms persist for months or even years, following medical treatment in roughly 35% of afflicted people, impairing everyday functioning, limiting the ability to return to work, and lowering the overall QOL. Recent studies point toward a key role of inflammatory pathways in the CRCI genesis. Attention to physical activity as a potential supportive care option is therefore increasing. However, evidence for the positive effects of exercise on preventing CRCI is still lacking.

**Patients and Methods:**

Against this background, the prospective, two-arm, 1:1 randomized, controlled trial investigates the influence of first line chemotherapy accompanied by exercise training on preventing CRCI in 126 patients with breast cancer at the local University Hospital. The study will evaluate biomarkers and secondary assessments suspected to be involved in the pathogenesis of CRCI in addition to objective (primary outcome) and subjective cognitive function. CRCI is believed to be connected to either functional and/or morphological hippocampal damage due to chemotherapy. Thus, cerebral magnetic resonance imaging (MRI) and hippocampal volume measurements are performed. Furthermore, a specific neuropsychological test battery for breast cancer patients has been developed to detect early signs of cognitive impairments in patients and to be integrated into practice.

**Discussion:**

This study will explore how a long-term supervised exercise intervention program might prevent CRCI, enables optimization of supportive care and objectifies limits of psychological and physical resilience in breast cancer patients during and after chemotherapy treatment.

**Trial Registration:**

ClinicalTrials.gov: Identifier: NCT04789187. Registered on 09 March 2021.

## Introduction

Despite the efficacy of breast cancer treatments, chemotherapy has severe side effects on patients' cardiovascular and metabolic systems and quality of life (QOL). Some adverse effects can be acute, occurring primarily during treatment, whereas others may have a delayed onset and persist for years after the end of treatment (1).

The variability of these side effects is widespread. Chemotherapy often leads to nausea, vomiting, depression, reduced bone-mineral density, cardiac toxicity, and cancer-related fatigue (2, 3).

Targeted exercise programs reduce several diseases and treatment-related side effects, including fatigue, depression, and lymphedema, mentioned above, besides increasing completion rate (4, 5). Therefore, exercise regimens can improve patients‘ QOL during and after therapy (6).

Cancer-related cognitive impairment (CRCI) – formerly termed as “Chemobrain” – can be observed before, during, and after treatment in breast cancer patients (7–9).

Mild to moderate deficiencies in learning, memorization, processing speed, and executive processes (10) are the most commonly reported impairments, and they significantly affect patients' QOL (11, 12). The percentage of breast cancer patients reporting cognitive impairments varies between 17 and 75% (13–15). This difference may be driven by varying assessment time points, assessment methods, criteria of cognitive impairment cut-offs, and various treatment/intervention regimens as well as interactions with correlating side effects, such as depression, and fatigue (13).

Although information about the underlying mechanisms of CRCI is still sparse. Investigations conducted in the past decade suggest a multifactorial genesis (16, 17).

Preclinical studies have shown that cytostatic drugs and radiation directly impair and damage different cell populations (e. g., neuronal progenitor cells and oligodendrocytes) (18) in the central nervous system (CNS). Clinical investigations, including microscopic and molecular findings, have revealed substantial reductions in gray and white matter volume and integrity (13, 19–26). These effects are not confined to treatments and drugs that can penetrate the blood-brain-barrier directly, suggesting mechanisms that are more complex.

According to recent literature, most likely indirect effects are disease- or therapy-related. They may translate to systemic inflammatory stimuli that penetrate the blood-brain-barrier causing functional and structural damage, and hence cognitive impairments (27). The role of systemic inflammation in this context has been accepted as a key player in the pathogenesis of neuropsychiatric diseases, and it demonstrates the delicate interplay between immune, nervous and central nervous systems in various pathological conditions (28).

Accomplishments in neuroscientific research conducted in humans and rodents have indicated that physical activity and exercise constitute a conservative and effective treatment method especially in psychiatric disorders such as major depression. Therefore, these activities have become a focus of interest for supportive therapies in oncology patients to prevent cognitive impairment (29–32).

Increased physical activity following diagnosis has been linked to primary cancer prevention and decreased mortality in oncology patients. Physical exercise interventions, therefore, became an integral and important part of supportive therapy in cancer patients (33), since they effectively reduce several side effects such as fatigue (34) and depressions as well as lymphedema and improve patient's fitness and QOL (35, 36).

Through a survey of the published literature, our group has found that exercise therapies considerably improve and preserve cognitive performance in healthy adults (27, 31, 37–39) and patients in clinical settings (40). Exercise appears to significantly reduce CRCI, according to preliminary findings. Preclinical studies have shown that physical activity reduces (neuro-) inflammatory stress in the long run and raises circulating and central levels of neuronal growth factors (41–47).

In this context, the most frequently investigated brain structure is the evolutionarily highly conserved hippocampus, which is involved in multiple cognitive activities such as learning, memory, and pattern recognition. It became a focus of interest in recent studies investigating CRCI, due to its sensitivity, ability to perform neurogenesis, and integration in various psychiatric and neurodegenerative disorders (37, 38, 48, 49).

Therefore, the majority of the findings mentioned above concern to the hippocampus, where the influence of physical exercise and neurogenesis has been associated with an increase in hippocampal volume and an enhanced memory function (31, 50, 51).

Regarding the type and intensity of the implemented exercise regimen, comprehensive reviews have shown that higher intensity exercise regimens may have superior effects on cognition, and they may be linked to physiological underpinnings compared to low or moderate aerobic exercise interventions (50, 52–59). In a randomized pilot trial Lee et al. (60), noticed that high-intensity interval training (HIIT) during chemotherapy was safe and feasible for women with breast cancer (60). Mijwel et al. (55) used HIIT in their training regimen demonstrated its efficacy in breast cancer patients undergoing chemotherapy (55). HIIT appears to be highly effective in minimizing side effects and is more efficient in improving functional cognitive impairments than an aerobic exercise intervention alone (36, 55–57).

As mentioned earlier, the symptom-complex related to CRCI is highly heterogeneous and varies substantially from patient to patient. Although, stated previously, the hippocampus is essentially involved in most of the CRCI related activities. We, therefore, use the California Verbal Learning Test (CVLT) to measure the primary endpoint of the conducted trial – neurocognition regarding learning, memorizing, processing speed, and executive functions because of its high sensitivity to hippocampal related impairments (61, 62). Brain magnetic resonance imaging (MRI) and hippocampal volumetry is used in conjunction with potential structural abnormalities (26, 63).

In addition, a specific neuropsychological test battery for breast cancer patients has been developed, which will help to specify and detect early signs of cognitive impairment in patients and will be integrated into a new standard of treatment.

Unfortunately, exercise trials in the context of CRCI have several methodological limitations. The majority of studies (59, 64–66) conducted to date were small in cohorts and time, were not randomized, used a variety of unstandardized objective and subjective cognitive assessments, provided no information on physiological/biological changes, and most essentially did not include imaging techniques to screen for structural correlates in terms of cognitive impairment. Incompliance and lack of adherence in the intervention group are common problems, also when seen to be correlating directly with the median age of study cohort, entity of cancer and especially the type and administration of the exercise intervention (supervised/unsupervised) (67, 68).

Therefore, the ECCO-study bridges the gap between conducted exercise trials by summarizing all findings to CRCI up to this time and integrating them into a randomized controlled trial.

The aim of this study is to investigate the effects of an additional 12 months individualized and supervised HIIT based training program, in contrast to only giving out general physical activity recommendations, on cognition in patients with diagnosed local breast cancer receiving neo-/adjuvant chemotherapy. Secondary aims are to determine the effects of the intervention on physical performance, common cancer related side effects (e.g., fatigue, emotional and physical well-being and QOL), structural changes of the brain, as well as potential underlying biomarkers.

Therefore, we hypothesize, that constant physical activity and additional supervised HIIT in the intervention group results in less cognitive impairment compared to the control group. Hence, the mean (sub)scores “total trial,” “short delay” and “long delay” of the CVLT after 12 months of intervention are hypothesized to be higher in the intervention- than in the control group.

## Methods

The Exercise, Cancer and Cognition (ECCO) study was designed as a randomized controlled trial to investigate the impact of a long-term physical activity program on CRCI in patients receiving adjuvant/neoadjuvant breast cancer treatment.

**Figure 1:**
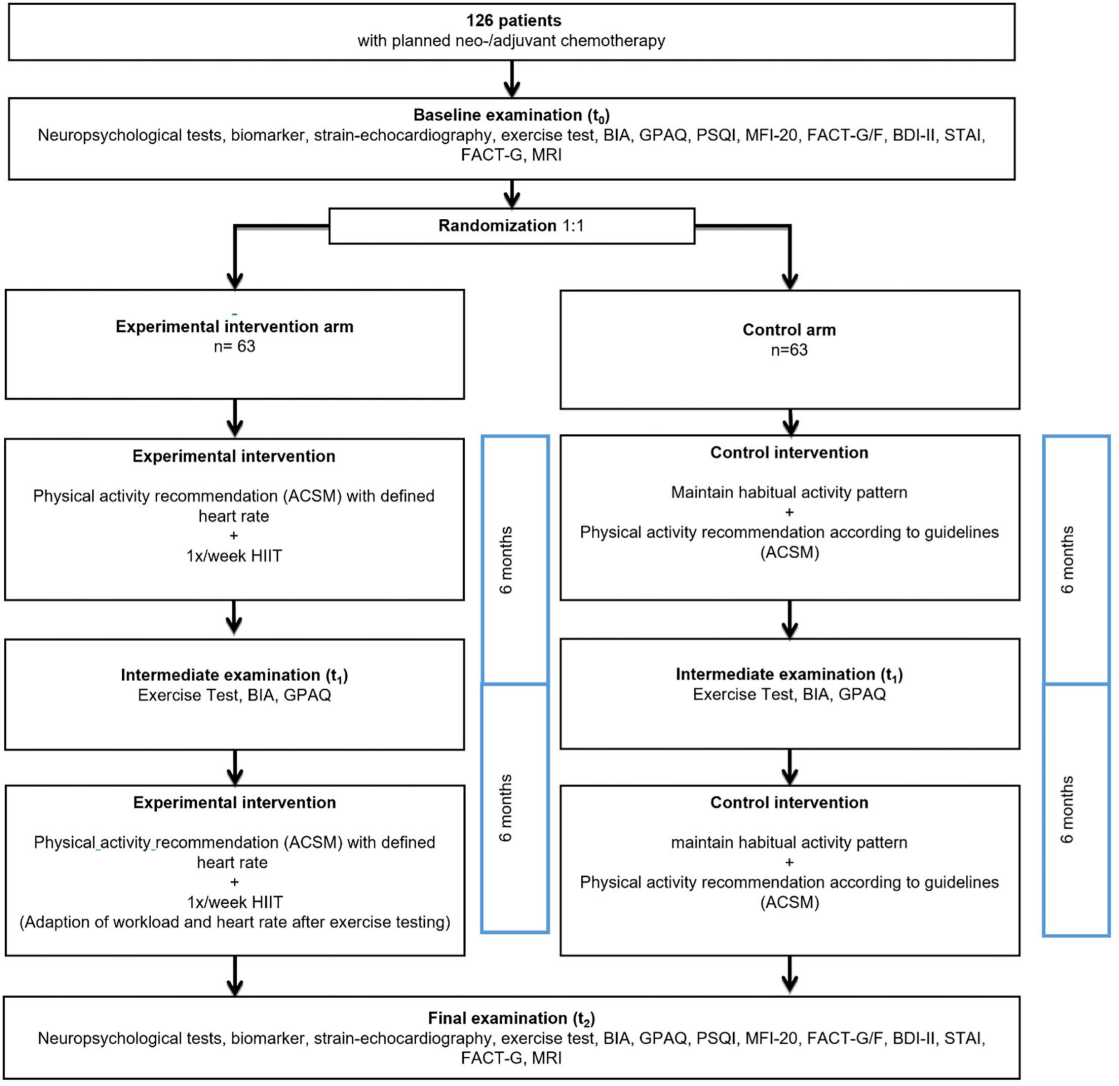
Study flow.

The ECCO study is a monocentric two-arm 1:1 randomized controlled trial (RCT), including a 12-month intervention and control group. A detailed study-flow is shown in Figure 1. The exercise interventions start within the first week of chemotherapy and last for 12 months. The study protocol was approved by the local ethics committee of Upper Austria (Austria) (1191/2018) and registered with the WHO trial register (reference number: NCT04789187). Any modifications to the protocol, which had an impact on the realization of the study, on benefits and harms of the participants, on study objectives and design, on sample size or study procedure, required a formal amendment to the Ethics Commission and had been approved. Participants are assigned to one of two groups: an exercise intervention arm (A) and control arm (B). Group A is motivated to fulfill the general physical activity recommendations (individual heart rate definition and strength training advice, home based) (69) and should perform a supervised high intensity interval training (HIIT, center based) once a week in addition to regular care. The control arm (B) receives regular care and general physical activity recommendations. Both groups will follow the same recommended physical activity guidelines, but in contrast to the intervention arm, the control group does not receive any individual heart rate suggestions and strength training material.

### Study Population and Recruitment

Patients are recruited at the department of hematology and internal oncology of the Kepler University Hospital in Linz (AUT). Patients diagnosed with local breast cancer receiving neo-/adjuvant chemotherapy and not fulfilling any exclusion criteria listed in Table 1 are advised by the attending physicians and recruited for study participation. Patients receive a detailed description of the study, can ask any question, and are included in the planned research program only after written consent, according to the ethics committee's approval. Patients can withdraw at any time and for any reason without prejudice to their medical care. Randomization procedures and randomization lists were deposited in a sealed envelope before recruitment of the first patient. Single decoding is available for each subject in sealed envelopes. The envelopes containing information about group affiliation are opened by an authorized study team member at the last possible time (at the earliest after complete baseline evaluation).

**Table 1:** In- and exclusion criteria.

**Inclusion criteria**	**Exclusion criteria**
• Pending chemotherapy (neo-/adjuvant)	• Significant comorbid conditions contraindicating physical activity
• Diagnosed breast cancer	• Cognitive dysfunction, defined by subjective or diagnostic deficits in attention, memory, executive function and/or visual construction before the start of chemotherapy, as well as any known history of cognitive disorder at the baseline screening.
	• In tests without clear ranges of reference values for cognitive impairments in performance, cut-offs will be defined using >1,5 SD from normative data. Cognitive impairment therefore is defined by > 1,5 SD in two tests at baseline assessment.
• Eastern Cooperative Oncology Group (ECOG) performance status of 0 or 1	• Vigorous physical activity of >300 min weekly within the last 6 months before diagnosis of breast cancer (Evaluated by training anamnesis)
• Age ≥ 18–70 years	• Patients unwilling to complete endurance exercise or complete all questionnaires related to the study
• Adequate hematologic function Platelet count > 50 × 10^∧^9/L Hemoglobin > 8 g/dL	• Past or current history of other malignant neoplasms other than breast cancer in the last 5 years
• No motorical dysfunction leading to the disability to perform endurance exercise according to protocol	• Coronary heart disease
• Signed informed consent prior to randomization	• Left bundle branch block
• Fluid in German	• Chemotherapy or radiation with other indications than breast cancer diagnosis
	• Current pregnancy or plans to become pregnant within the next 3 years
	• Neurodegenerative disease

All assessments are conducted at three main time points (t_0_, t_1_, t_2_) in both groups.

- t_0_ (Pre): Before first chemotherapy within ± 14 days.
- t_1_ (interim): After 6 months ± 14 days.
- t_2_ (post): After 12 months ± 14 days (end of intervention).

For participants in all groups, clinical assessments during the intervention period are additionally performed at least every second month and at each clinical visit (usual care).

### Data Collection and Timeline/Testing Procedure

Data are collected at baseline (t_0_), after 6 months of intervention (t_1_) and after 12 months of intervention (t_2_). The patient's medical record and intended therapy, demographic (age, sex) and anthropometric data (weight, height, BMI), past medical history and current medication are collected at the start. Furthermore, the primary outcome and all secondary outcome measurements of the study are captured before the first therapy session. After 6 months of intervention (t_1_), an interim exercise test is conducted to adapt the exercise workload in Arm A and in Arm B for equality. At (t_2_) the primary outcome measures and all secondary outcome measures are re-assessed. A follow-up period of 12 and 24 months is provided after the end of the intervention. The follow-up assessments therefore are parameters assessed within the clinical routine amended by the questionnaires used to assess quality of life and activity level.

A detailed study schedule is presented in Supplementary Appendix 1.

All assessments except exercise testing are performed at the Kepler University Hospital, Linz (AUT). At (t_0_), (t_1_), and (t_2_). All blood samples are drawn and stored at minus 85°C at the Kepler University Hospital Linz. Analyses of cancer-related pro- and anti-inflammatory biomarker samples are performed at the Sports University of Cologne.

### Exercise Testing

Incremental exercise tests with a one-minute step duration are conducted on a calibrated bicycle ergometer (Corival cpet, Lode BV Medical Technology, NL) (70). Before each test, the breathing gas analyser (Vyntus CPX, CareFusion Germany 234 GmbH, D) is calibrated for volume and O_2_/CO_2_ concentration according to the manufacturer's guidelines. Every participant is seated for about 1–2 min on the ergometer while preparing one ear lobe for blood-testing. During exercise tests, respiratory gas exchange is recorded, and heart rate is measured continuously using the ECG system KISS MULTILEAD (GE Medical Systems Information Technologies GmbH, D). Lactate concentrations are determined in capillary blood taken from the ear lobe at rest and at the end of each increment until exhaustion and therefore end of testing.

### Groups and Description of Intervention

#### Exercise Intervention Arm (A)

Participants randomized to the exercise arm are motivated to fulfill the general physical activity recommendations with a defined heart rate and material for strength training (folder and exercise video). The frequency and duration of the home-based exercise depends on individual circumstances but follows general suggestions.

American College of Sports Medicine (ACSM) has recommended the following physical activity for cancer survivors (69):

- Take part in regular physical activity.
- Avoid inactivity and return to normal daily activities as soon as possible after diagnosis.
- Aim to exercise at least 150 min weekly with moderate intensity.
- Include strength training exercises at least 2 days per week.

The advised heart rate therefore is defined by the baseline exercise testing and adjusted after 6 months if necessary.

Additionally, group A is asked to perform a supervised HIIT at the Cardiomed Linz rehabilitation center once a week.

The high intensity exercise interventions are performed on a stationary bike (ergo_bike premium 8, daum electronic gmbh, D). An experienced and accredited therapy specialist supervises and supports the exercise interventions throughout the trial.

Participants perform alternating intervals of maximal power output P_max_ (measured by the exercise test) and rest in a ratio of 1:3. The duration of the P_max_ interval is 20 and 60 s for pause.

**Figure 2:**
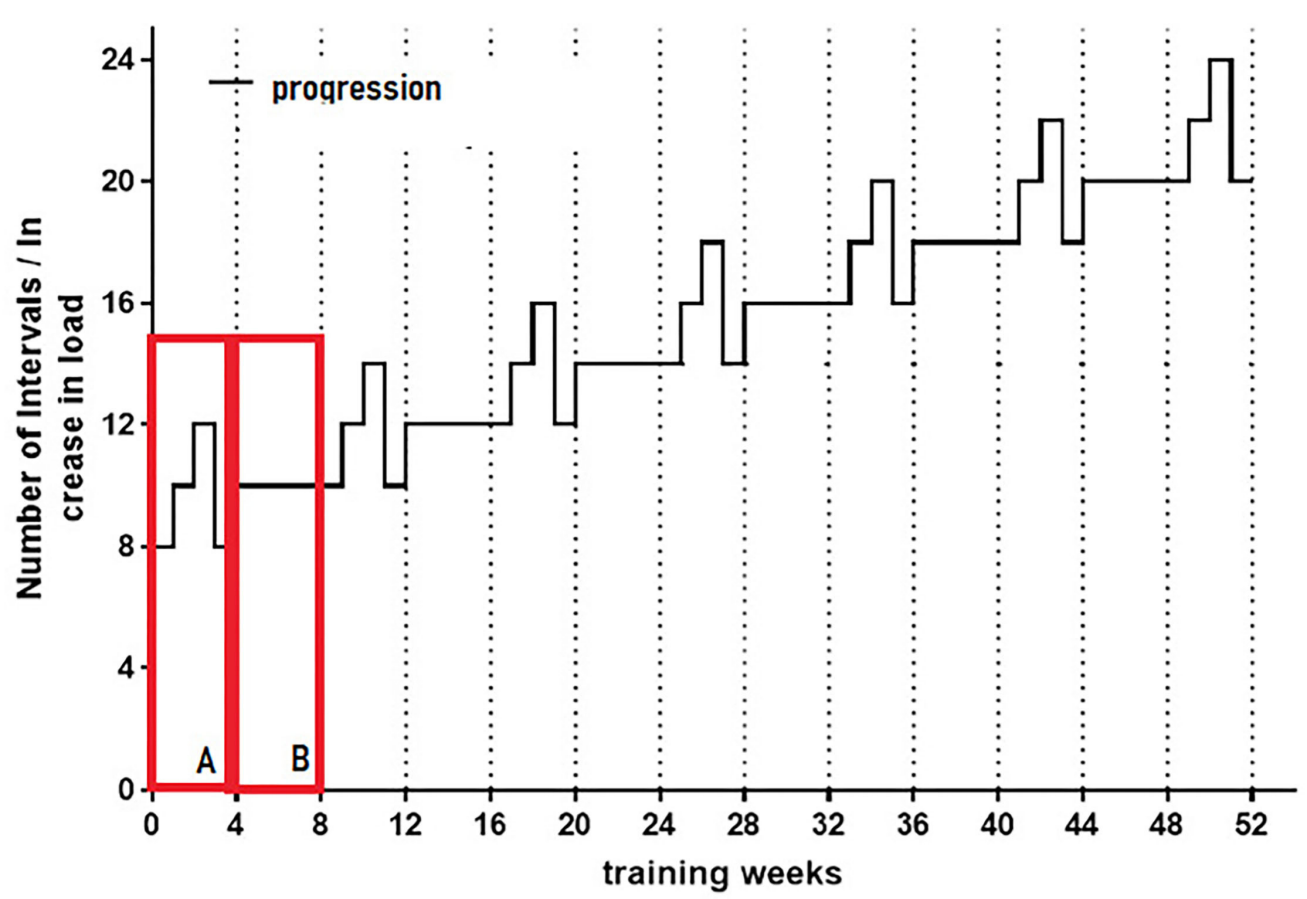
Schematic representation of the amount of intervals for the first 6 month of intervention. HIIT is planned in 2 cycles, **(A)** Continual enhancement of the amount of intervals up to week 3 followed by one recovery week (3:1). **(B)** Stabilization cycle, which includes 4 weeks of a continual amount of intervals. Those stabilization cycles will be enhanced over time by the amount of intervals. This cyclic scheme will be maintained over the year of intervention period.

The HIIT program is set up in two cycles; one includes an enhancement of the weekly intervals and a recovery unit (3:1) (shown in Figure 2A). This cycle is followed by a stabilization cycle (4 weeks), in which the number of intervals is kept constant (shown in Figure 2B). The next cycle starts with a higher number of intervals and is enhanced over 3 weeks, followed by a recovery unit (3:1), and continued by another stabilization cycle (4 weeks). A schematic graphic of this periodized exercise regimen is shown in Figure 2 (71). After 6 months of intervention, a re-evaluation of the patient's performance is conducted as described above. The peak load of the intervals will be adapted according to the new P_max_ if any alteration in peak performance is observed.

**Figure 3:**
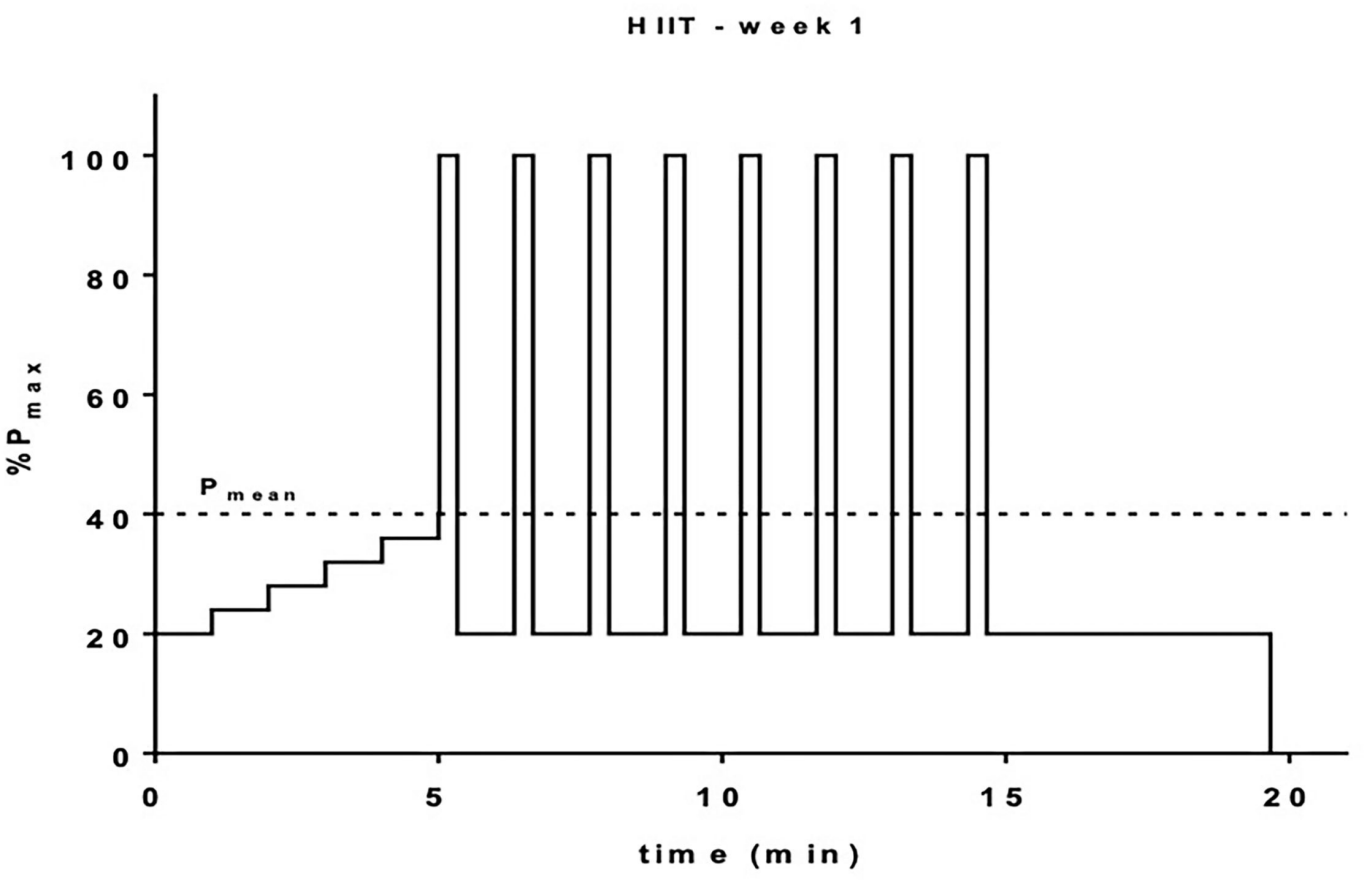
Schematic representation of the HIIT for the first week of intervention. Warm-up exercise starts low at 20% P_max_ and will be increased in 5 increments up to 40% P_max_. Intervals will be performed at 100% P_max_ for 20 s and recovery intervals will be 60 s at 20% P_max_ giving a mean workload (P_mean_) of 40% P_max_ according to Tschakert and Hofmann (QUELLE). Overall workout duration is 20 min.

HIIT in week 1 is conducted with 8 intervals (see Figure 3) and enhanced to 10 intervals in week 2 and 12 in week three. The recovery units are adapted to the number of intervals. The following stabilization cycle includes 10 intervals over 4 weeks. The duration of the HIIT sessions therefore depends on the amount of intervals and ranges from 20 min at the beginning of the program to ~45 min at the end of the intervention.

HIIT load is enhanced over time by increasing the amount of intervals and increased to a 2:1 ratio (rest to load) in the last 3 months, according to expected training adaptations.

#### Control Arm (B)

Participants in the control arm receive usual care and physical activity recommendations following regular procedures. Patients are examined during clinical visits. The control group receives the same physical activity recommendations (see above) as the intervention group except any heart rate suggestions.

#### Documentation of Exercise Intervention

Center based exercise interventions in arm A and all home-based physical activity (arm A and B) are recorded by a “Garmin Forerunner 45s” (with “Garmin Connect”) in combination with a chest strap. Heart rate, frequency, and duration of home exercises are documented in a digital activity diary provided on the online platform “www.trainingpeaks.com”. Patients perform low intensity endurance exercise at home with any type of sports equipment or outdoors independently.

### Study Endpoints

#### Primary Endpoint

The primary endpoint is the difference from baseline (t_0_) to completion of the exercise intervention (t_2_), in the California Verbal Learning Test (CVLT) mean scores and consists of the three (sub)scores “short-delay,” “long-delay” and “total-trials 1–5” of the CVLT. The CVLT is an instrument commonly used to evaluate the individual verbal learning and memory.

The test consists of two 16-word lists. The word list A is defined as the learning-list; word list B is used to interfere (interference-list). Word list A will be read aloud to the patient 5 times, then list B will read once. After that, the patients must recall as many words as possible from list A, afterwards the semantic recall follows. After 20 min, the patient is asked to recall again as many words as possible from list A, followed by a helping recall trail. For recalling, similar, semantic and phonematic distractions and categoric prototypes will be used. There are two normed settings available for baseline and t_2_.

The (sub)scores “total-trials 1–5” ascertains the learning curve for the first word list based on 5 rounds. The “short-delay” reports the delayed recall immediately after presentation and retrieval of the second word list (as interference) and without repeating the first list. The “long-delay” is testing the recall after ~20 min.

#### Secondary Endpoints

Further neuropsychological related endpoints distinguish by the variable attention and motor-speed, memory, executive functions, and visual construction.

To raise attention and psycho-motoric speed the subtest Digit Symbol Coding (of WAIS IV) is used, in which as fast as possible, symbols shall be written to different objects and the Trail Making Test A, in which numbers shall be put together in the correct order.

Memory function is captured with the subtests Logical Memory I & II (of WMS IV, memorizing texts immediately and after ~30 min). The subtest Digit Span backwards (of WAIS IV), Trail Making Test B/A (quotient of the processing times required for Trail Making Test B) and the “Regensburger-Wortflüssigkeits-Test” for word fluency (words with a special initial letter, words from a special category) measure executive functions.

At last, the subtest Block Design (of WAIS IV) in which patterns have to be recreated from a template with cubes is used to get information about the visual construction.

Secondary endpoints of physical activity and exercise related aspects are represented by performance changes obtained from exercise testing. Main variables are maximal oxygen uptake (VO_2max_) and maximal power output (P_max_), submaximal threshold values such as the first and the second ventilatory thresholds (VT_1_, VT_2_), body composition (body fat, muscle mass, Waist-to-Hip ratio, BMI) and the global physical activity questionnaire (GPAQ).

Additionally morphological changes of the heart will be objectified by strain-analyses. Speckle-tracking-echocardiography performed by Aplio-i900 (Canon) is implemented to evaluate possible chemotherapy related effects on the ventricular function.

Further patient-related endpoints like fatigue, emotional functioning/well-being, physical functioning/well-being, and QOL are measured by the Pittsburgh Sleep Quality Index (PSQI), Multidimensional fatigue inventory (MFI-20), Functional Assessment of Cancer Therapy-General (FACT-G, FACT-G/F), Beck-Depression-Inventar (BDI-II) and State-Trait-Anxiety Inventory (STAI).

Survival related endpoints are defined as the time from randomization to any signs of locoregional or metastatic recurrence or the appearance of secondary cancer or death, whichever occurs first - disease free survival (DFS) and time from randomization to death from any cause - overall survival (OS).

Endpoints of cerebral imaging are measured by Magnetic Resonance Imaging (MRI) and analyzed by “FreeSurfer” to show morphological signs of chemotherapy related effects on hippocampus volume. Cerebral images are acquired by a 3-Tesla Magnetom Trio Scanner (Siemens, Germany). For volume hippocampal measurement the automated volume-measurement software “FreeSurfer,” V.6.0 is used.

Laboratory endpoints, former connected to CRCI, are measured by pro- and anti-inflammatory biomarkers including Tumor necrosis factor alpha (TNF-α), Interleukin1 alpha,−1 beta,−6,−10 (IL-1α, IL-1β, IL-6, IL-10), C-reactive protein (CRP), Interleukin-1 receptor antagonist, (IL-1RA) and Neurotrophic and growth factors including Brain-derived neurotrophic factor (BDNF), Insulin like growth factor (IGF-1), Vascular Endothelial Growth Factor (VEGF) and Matrix-Metalloproteases (MMP). The primary endpoint and all secondary endpoints are summarized in Table 2.

**Table 2:** Primary and secondary endpoints.

**Primary endpoints**
California verbal learning test • “Total trials 1–5” • “Short delay” • “Long delay”
**Secondary endpoints**
Neuropsychological endpoints:
Attention
• Wechsler Adult Intelligence Scale (WAISIV) – Subtest Digit Symbol Coding
• Trail Making Test A
Memory:
• Wechsler Memory Scale (WMS-IV)
• Wechsler Adult Intelligence Scale (WAIS-IV) - Subtest Logical Memory I & II, Digit Span
Executive function:
• “Regensburger” Wortflüssigkeits-Test (RWT)
• Trail Making Test B/A
• Wechsler Adult Intelligence Scale (WAIS-IV) - Subtest Digit Span backwards
Visual construction:
• Wechsler Adult Intelligence Scale (WAIS-IV) - Subtest Block Design
Cerebral imaging endpoints
• MRI and multiparametric automated volumetric analysis by FreeSurfer with respect volumes, cortical surface deformation, segmentation of the white matter and deep gray matter structures, as well as the shift in tissue intensity
Laboratory endpoints
Pro- and anti-inflammatory biomarker • Tumor necrosis factor alpha (TNF-α) • Interleukin1 alpha,−1 beta,−6,−10 (IL-1α, IL-1β, IL-6, IL-10) • C-reactive protein (CRP) • Interleukin-1 receptor antagonist, (IL-1RA)
Neurotrophic and growth factors • Brain-derived neurotrophic factor (BDNF) • Insulin like growth factor (IGF-1) • Vascular Endothelial Growth Factor (VEGF) • Matrix-metalloproteases (MMP)
Physical activity and Exercise Endpoints
• Exercise testing • Body-Mass Index (BMI) • Bio Impedance Analysis (BIA) • Hip-to-Waist ratio (HtW-R) • Global physical activity questionnaire (GPAQ)
Cardiac imaging Endpoints
• Speckle tracking strain echocardiography
Survival related Endpoints
• Disease free survival and overall survival
Patient related Endpoints
• Pittsburgh Sleep Quality Index (PSQI) • Multidimensional fatigue inventory (MFI-20) • Functional Assessment of Cancer Therapy-General (FACT-G, FACT-G/F) • Beck-Depression-Inventar (BDI-II) • State-Trait-Anxiety Inventory (STAI)

### Drop-Out Procedure

Patients can quit study participation at any time and for any reason. Except for personal reasons, participation is terminated if participants miss HIIT sessions for more than five weeks in a row. In order to collect sufficient data for statistical analysis, participants are asked to undergo all endpoint measurements at the time of study drop-out.

### Adverse Events Management

Neither additional nor experimental drugs are given to patients during this clinical trial. Patients in the study arm are asked to follow an individual training regimen based on the most recent health guidelines and so did not pose a health risk. The included procedures are validated and performed under the supervision of a physician.

### Data Management

Clinical research and management software (REDCAP^®^) is used to collect the data electronically. To protect the confidentiality of the participants, all required information is identified by an individual ID number. Separate electronic case report forms (eCRF) are used for baseline and follow-up of structured interviews with study participants. In case of technical failure, paper case report forms (CRF) are used. When available, values of laboratory tests and additional data are added to the electronic database. All data capture is pre-specified by the principal investigator.

### Biostatistical Planning and Analysis

#### Concept

The study is based on a superiority approach with a comparison of two kinds of treatment and with three equal-ranking primary endpoints.

Treatment arms:

Treatment arm A: Endurance exercise intervention → group A
Treatment arm B: No endurance exercise intervention → group B

Primary endpoints:

(1): CVLT total trials 1-5 after 12 months [-]
(2): CVLT short delay after 12 months [-]
(3): CVLT long delay after 12 months [-]

The type I error will be adjusted according to Bonferroni.

#### Hypotheses

(1):

H-01:
CVLT total trials 1–5 after 12 months is not higher in group A than in group B
H-11:
CVLT total trials 1–5 after 12 months is higher in group A than in group B

(2):

H-02:
CVLT short delay after 12 months is not higher in group A than in group B
H-12:
CVLT short delay after 12 months is higher in group A than in group B

(3):

H-03:
CVLT long delay after 12 months is not higher in group A than in group B
H-13:
CVLT long delay after 12 months is higher in group A than in group B

### Sample Size Estimations and Determination of Sample Size

The following information and assumptions are used for sample size estimations:

- Use of data from the study of Andreotti et al. (72) (baseline assessments in healthy women) for group A.
- Postulation of a 15% change of the mean scores in group A over group B for all 3 primary endpoints (Xgroup A = 1.15 ^*^ Xgroup B).
- Postulation of a 7.5% change of the standard deviations in group A over group B for all 3 primary endpoints (SDgroup A = 1.075 ^*^ SDgroup B).
- Type I error (Bonferroni-adjusted) = 0.833% one-sided.
- Type II error = 20%.
- Independent samples *t*-test.

#### Results of Sample Size Estimations

The result of the sample size estimation (see Table 3) is a requirement of 48 “valid cases” per group. According to previous work and our clinical expertise we calculated sample size with *n* = 126, expecting a drop out of 15%. As drop-out rates in the literature often show great variety and the applied exercise regime (even though it is highly time efficient and supervised) is rather newly tested within this group, study capacities and all arising costs will be fully covered if we see higher drop-out rates up to *n* = 134 (Assuming a drop-out rate of 20% and considering the possible need for a non-parametric test).

**Table 3:** Sample size estimation.

	**Group B**	**Group A**	**Delta**	**Pooled SD**	***n***
CVLT total trials 1–5 after 12 months	48.56 ± 7.47	55.84 ± 8.03	7.28	7.75	2 × 25
CVLT short delay after 12 months	11.02 ± 2.26	12.67 ± 2.43	1.65	2.35	2 × 44
CVLT long delay after 12 months	10.97 ± 2.38	12.62 ± 2.56	1.65	2.47	2 × 48

### Analysis Populations

#### Intent-To-Treat (ITT) Population

All subjects whose study intervention has actually started (group A) or who have reached a time when the study intervention could have started (group B) will be included in the ITT population. All variables will be analyzed.

#### Per-Protocol (PP) Population

All subjects without occurrence of any drop-out situation (all valid cases) will be included in the PP population. All variables will be analyzed. The PP analysis is paramount.

#### Full Analysis Set

All enrolled subjects, that is the ITT population plus subjects whose study intervention has never started (group A) or who don't have reached a time when the study intervention could have started (group B). All variables of all subjects not belonging to the ITT population are analyzed by descriptive statistics, provided that the number of the corresponding subjects is higher than 5, otherwise a narrative presentation of these cases takes place.

#### Handling of Implausible Values and of Missing Values

Implausible values have to be identified during the data management process in agreement with the clinical investigator. They will be converted into missing values.

Missing values will be replaced only in the ITT population and only for the three primary endpoints. The replacements will be made according to the worst-case principle (use of the worst assessed value in the study).

### Statistical Methods

#### Group Comparisons of Primary Endpoints/Hypotheses Testing

If the hypothesis of normal distribution cannot be rejected (Kolmogorov-Smirnov with Lilliefors significance correction, type I error = 10%) and if homoscedasticity exists (Levene's test; type I error = 5%) a parametric analysis of covariance (ANCOVA) will be applied (type I error = 0.833% one-sided). Otherwise, a non-parametric analysis of covariance (rank analysis of covariance according to Quade) will be used (type I error = 0.833% one-sided).

Covariates are

- CVLT total trials 1–5/CVLT short delay/CVLT long delay (corresponding variable) at baseline [-]
- BMI at baseline [kg/m^2^]
- Age at baseline [years]
- BDI II at baseline [-]
- STAI at baseline [-]
- Fatigue at baseline [yes/no]

#### Group Comparisons of Further Variables

All other variables will be analyzed by usual parametric and non-parametric tests for univariate comparisons of independent samples.

#### Correlation Analyses

Parametric and non-parametric correlation coefficients will be calculated to test relationships between the primary endpoints and other outcome variables.

#### Regression Analyses

The influence of exercise and exercise test results on overall survival and disease-free survival will be investigated by cox regression analyses. Multiple regression analyses will be used to investigate the influence of baseline characteristics and any specific findings during the intervention phase on the primary endpoints in group A.

#### Two-Sided 95% Confidence Intervals

For selected variables, two-sided 95% confidence intervals will be calculated.

#### Type I Error Adjustment

Except for hypotheses testing no adjustment for the type I error will be made. Therefore – apart from hypotheses testing – the results of inferential statistics will be descriptive only.

### Presentation of the Results (Descriptive Analysis, Graphs etc.)

Categorical variables will be presented using counts and percentages. Variables measured on ordinal scales will be presented using counts and percentages (where appropriate) or minimum, 25%-percentile, median, 75%-percentile, maximum and number of patients (where appropriate). Continuous variables will be presented using minimum, 25%-percentile, arithmetic mean, median, 75%-percentile, maximum, standard deviation, and number of patients.

All results will be presented in the form of tables, selected results additionally in the form of graphs (bar charts, boxplots).

Time-to-event variables will be depicted by Kaplan Meier plots.

### *Post-hoc* Analyses

Analyses of subgroups and other *post-hoc* analyses (e.g., further correlation and regression analyses or analyses of covariance) can be performed for cause. However, all statistical results will be only descriptive.

## Interim Analyses

A priori no interim analysis is intended.

## Randomization

Randomization procedures and randomization lists are deposited in a sealed envelope. An opening is only allowed after completion of the entire study (in case of scheduled study termination after notification of the completion of all data entries and checks by the biostatistician, in case of premature study termination after obtaining the permission of the biostatistician).

Single decoding is available for each subject in sealed envelopes. The envelopes containing information about group affiliation will be opened by an authorized study team member at the last possible time (at the earliest after complete baseline evaluation).

## Discussion

The ECCO trial investigates the effects of a personalized home-based physical activity and supervised HIIT on the prevention of CRCI in breast cancer patients undergoing neo-/adjuvant chemotherapy. To the best of our knowledge, it is the first “long-term” RCT, which includes a comprehensive set of behavioral, biological, and imaging measurements. As a result, this study is a unique and promising attempt to learn more about the underlying mechanisms of CRCI and exercise as a potential treatment strategy to avoid cognitive side effects. Another important factor relates to the dose and frequency of the exercise interventions, and the duration of the program. While the time of intervention in comparing studies ranges from 3 weeks to 6 months (17) and have been discussed to be too short in their design related to investigate on cognitive endpoints, the present study accompanies the targeted patients for a total intervention period of 12 months.

However, there are some unanswered questions regarding exercise intervention on preventing CRCI. Previous studies have shown that HIIT is viable in breast cancer patients (27). A higher-intensity exercise load may have better effects on cognition and physiological processes than moderate exercise training alone (27). This randomized controlled trial, therefore, applies structured home-based physical activity and a well-structured supervised weekly HIIT intervention.

Understanding immunological and neurological pathways that may underpin CRCI is still in its infancy. Physical activity appears to affect immunological and neuroprotective processes (71). Exercise also lowers resting levels of inflammatory markers in women with breast cancer (72). Despite the lack of data, this study investigates the promising hypothesis that inflammatory processes and neurotrophic factors are the underlying mechanisms that cause cognitive impairment. As a result, there are still a lot of limitations when it comes to different exercise modalities (frequency, intensity, type, and time – FITT) and the potential impacts and modulations of underlying biomolecular pathways in patients with breast cancer, for example.

More randomized studies are needed to investigate the link between exercise and neurocognition, chemotherapy side effects, cardiopulmonary fitness, emotional status, overall survival, and the morphological and structural elements involved in CRCI.

In conclusion, this research focuses on the most up-to-date exercise intervention, HIIT, demonstrated to have the best results in prior studies (54, 66). Another distinguishing feature is the 12 months duration of the intervention, which begins during chemotherapy. As defined by the three subcategories of the CVLT, the major endpoint can after further analyses be compared with hippocampus volume measurement, allowing this study to look at the morphological component, which others have studied (31, 50, 51). In addition to major goals, well-structured comprehensive methods are used to characterize the processes and effects of CRCI in breast cancer patients with apparent cognitive deficits. The involvement of these elements in the development and treatment of CRCI may be linked to structural and morphological changes in the hippocampal volume, (neuro-) inflammatory blood indicators, and neurotrophic growth factors.

We examine the underlying physiological mechanisms of cancer related cognitive impairments and how a well-structured and precisely defined prescribed training regimen including HIIT could help to avoid these effects. These parameters of training are still unknown in supportive care methods in relation to breast cancer patients who are undergoing chemotherapy.

## Ethics Statement

This study was conducted in accordance with the Statement on the Declaration of Helsinki and Good Scientific Practice following accepted ethical, scientific, and medical standards that protect the rights of participants. Ethical approval has been obtained from the Ethics Commission of Upper Austria, of the Kepler University Hospital (Austria) (reference number: 1191/2018). The trial was registered at ClinicalTrials.gov: Identifier: NCT04789187. Patients will be informed about the possible risks and benefits of the study. Participation in this study will be voluntary. Written informed consent will be obtained from all patients.

## Author Contributions

DK, MK-S, DF, PZ, MV, RK, SB, PH, and HO have been responsible for the development of the trial. MK-S, DK, and PH have written the manuscript for the study protocol with support of PZ, MV, JK, HB, KA, HH, and CT. All authors read and approved the final manuscript.

## Funding

Krebshilfe Oberösterreich - This research is mainly sponsored by the Austrian Krebshilfe - OÖ. Förderpreis Onkologie of the ÖGHO - In part this research was also sponsored by a research grant of the Austrian Society of Hematology and Medical Oncology.

## Conflict of Interest

The authors declare that the research was conducted in the absence of any commercial or financial relationships that could be construed as a potential conflict of interest.

## Publisher's Note

All claims expressed in this article are solely those of the authors and do not necessarily represent those of their affiliated organizations, or those of the publisher, the editors and the reviewers. Any product that may be evaluated in this article, or claim that may be made by its manufacturer, is not guaranteed or endorsed by the publisher.

## Glossary

ACSM: American College of Sports Medicine
AE: Adverse Event
ANC: Absolute Neutrophil Count
BDI: Beck Depression Inventory
BDNF: Brain-Derived Neurotrophic Factor
BMI: Body Mass Index
CAT: Computerized Adaptive Test
CEA: Carcinoembryonic AntigenCNS, Central Nervous System
CRA: Clinical Research Associate
CRCI: Cancer Related Cognitive Impairment
CRP: C-Reactive Protein
CVLT: California Verbal Learning Test
BC: Breast CancerBIA, Body-Impedanz-Analysis
CT: Computer Tomography
DCR: Data Clarification Request
DFS: Disease Free Survival
ECG: Electrocardiography
ECOG: Eastern Cooperative Oncology Group performance status
EF: Emotional Functioning
EORTC: European Organization for Research and Treatment of Cancer
EOS: End of Study
FA: Fatigue
FITT: Frequency, Intensity, Time, and Type
FPI: First Patient In
FU: Follow-Up
HIIT: High Intensity Interval Training
HR: Heart Rate
ICER: Incremental Cost Effectiveness Ratio
IEC: Independent Ethics Committee
IGF-1: Insulin Like Growth Factor 1
IGF: Insulin-like Growth Factor
IL: Interleukin
IL-1RA: Interleukin-1 Receptor AntagonistKcal, Kilocalories
LPO: Last Patient Out
MMP: Matrix Metalloproteases
MRI: Magnetic Resonance Imaging
MV: Months VisitNHS, Nurses Health Study
NYHA: New York Heart Association
OS: Overall SurvivalPA, Physical Activity
PF: Physical Functioning
PROs: Patient-Reported Outcomes
RFS: Relapse Free Survival
QALYs: Quality of Adjusted Life Years
QOL: Quality of Life
SAE: Serious Adverse Event
SOP: Standard Operating Procedure
STAI: State-Trait-Anxiety Inventory
TNF-alpha: Tumor Necrosis Factor Alpha
VEGF: Vascular Endothelial Growth Factor
W: Watt
WtHR: Waist to Hip Ratio
WI: Working instruction

## References

[1] Padegimas A Clasen S Ky B. Cardioprotective strategies to prevent breast cancer therapy-induced cardiotoxicity. Trends Cardiovasc Med. (2020) 30:22–8. 10.1016/j.tcm.2019.01.006 30745071 PMC7287268

[2] Galizia D Milani A Geuna E Martinello R Cagnazzo C Foresto M . Self-evaluation of duration of adjuvant chemotherapy side effects in breast cancer patients: a prospective study. Cancer Med. (2018) 7:4339–44. 10.1002/cam4.1687 30030895 PMC6144000

[3] Delgado-Ramos GM Nasir SS Wang J Schwartzberg LS. Real-world evaluation of effectiveness and tolerance of chemotherapy for early-stage breast cancer in older women. Breast Cancer Res Treat. (2020) 182:247–58. 10.1007/s10549-020-05684-5 32447595

[4] Sanft T Harrigan M Cartmel B Ferrucci LM Li FY McGowan C . Effect of healthy diet and exercise on chemotherapy completion rate in women with breast cancer: The Lifestyle, Exercise and Nutrition Early after Diagnosis (LEANer) study: Study protocol for a randomized clinical trial. Contemp Clin Trials. (2021) 109:106508. 10.1016/j.cct.2021.106508 34274495 PMC10424280

[5] Mijwel S Bolam KA Gerrevall J Foukakis T Wengström Y Rundqvist H. Effects of Exercise on Chemotherapy Completion and Hospitalization Rates: The OptiTrain Breast Cancer Trial. Oncologist. (2020) 25:23–32. 10.1634/theoncologist.2019-0262 31391297 PMC6964125

[6] Lahart IM Metsios GS Nevill AM Carmichael AR. Physical activity for women with breast cancer after adjuvant therapy. Cochrane Database Syst Rev. (2018) 1:CD011292. 10.1002/14651858.CD011292.pub2 29376559 PMC6491330

[7] Floyd R Dyer AH Kennelly SP. Non-pharmacological interventions for cognitive impairment in women with breast cancer post-chemotherapy: a systematic review. J Geriatr Oncol. (2021) 12:173–81. 10.1016/j.jgo.2020.05.012 32536427

[8] González-Santos Á Postigo-Martin P Gallart-Aragón T Esteban-Cornejo I Lopez-Garzon M Galiano-Castillo N . Neurotoxicity prevention with a multimodal program (ATENTO) prior to cancer treatment versus throughout cancer treatment in women newly diagnosed for breast cancer: Protocol for a randomized clinical trial. Res Nurs Health. (2021) 44:598–607. 10.1002/nur.22136 33963594

[9] Strandberg E Vassbakk-Svindland K Henriksson A Johansson B Vikmoen O Kudrén D . Effects of heavy-load resistance training during (neo-)adjuvant chemotherapy on muscle cellular outcomes in women with breast cancer. Medicine (Baltimore). (2021) 100:e24960. 10.1097/MD.0000000000024960 33725859 PMC7969308

[10] Ahles TA Root JC Ryan EL. Cancer- and cancer treatment-associated cognitive change: an update on the state of the science. J Clin Oncol. (2012) 30:3675–86. 10.1200/JCO.2012.43.0116 23008308 PMC3675678

[11] Selamat MH Loh SY Mackenzie L Vardy J. Chemobrain experienced by breast cancer survivors: a meta-ethnography study investigating research and care implications. PLoS ONE. (2014) 9:e108002. 10.1371/journal.pone.0108002 25259847 PMC4178068

[12] Ah D. von, Habermann B, Carpenter JS, Schneider BL. Impact of perceived cognitive impairment in breast cancer survivors. Eur J Oncol Nurs. (2013) 17:236–41. 10.1016/j.ejon.2012.06.002 22901546

[13] Janelsins MC Kesler SR Ahles TA Morrow GR. Prevalence, mechanisms, and management of cancer-related cognitive impairment. Int Rev Psychiatry. (2014) 26:102–13. 10.3109/09540261.2013.864260 24716504 PMC4084673

[14] Myers JS Erickson KI Sereika SM Bender CM. Exercise as an intervention to mitigate decreased cognitive function from cancer and cancer treatment: an integrative review. Cancer Nurs. (2018) 41:327–43. 10.1097/NCC.0000000000000549 29194066 PMC5975081

[15] Wefel JS Kesler SR Noll KR Schagen SB. Clinical characteristics, pathophysiology, and management of noncentral nervous system cancer-related cognitive impairment in adults. CA Cancer J Clin. (2015) 65:123–38. 10.3322/caac.21258 25483452 PMC4355212

[16] Jansen CE Cooper BA Dodd MJ Miaskowski CA. A prospective longitudinal study of chemotherapy-induced cognitive changes in breast cancer patients. Support Care Cancer. (2011) 19:1647–56. 10.1007/s00520-010-0997-4 20820813

[17] Zimmer P Baumann FT Oberste M Wright P Garthe A Schenk A . Effects of exercise interventions and physical activity behavior on cancer related cognitive impairments: a systematic review. Biomed Res Int. (2016) 2016:1820954. 10.1155/2016/1820954 27144158 PMC4842032

[18] Brown T McElroy T Simmons P Walters H Ntagwabira F Wang J . Cognitive impairment resulting from treatment with docetaxel, doxorubicin, and cyclophosphamide. Brain Res. (2021) 1760:147397. 10.1016/j.brainres.2021.147397 33705788 PMC9179831

[19] Seigers R Schagen SB van Tellingen O Dietrich J. Chemotherapy-related cognitive dysfunction: current animal studies and future directions. Brain Imaging Behav. (2013) 7:453–9. 10.1007/s11682-013-9250-3 23949877

[20] Han R Yang YM Dietrich J Luebke A Mayer-Pröschel M Noble M. Systemic 5-fluorouracil treatment causes a syndrome of delayed myelin destruction in the central nervous system. J Biol. (2008) 7:12. 10.1186/jbiol69 18430259 PMC2397490

[21] Dietrich J Han R Yang Y Mayer-Pröschel Margot Nobel Mark. CNS progenitor cells and oligodendrocytes are targets of chemotherapeutic agents in vitro and in vivo. J Biol. (2006) 5:22. 10.1186/jbiol50 17125495 PMC2000477

[22] Ahles TA Root JC. Cognitive effects of cancer and cancer treatments. Annu Rev Clin Psychol. (2018) 14:425–51. 10.1146/annurev-clinpsy-050817-084903 29345974 PMC9118140

[23] McDonald BC Saykin AJ. Alterations in brain structure related to breast cancer and its treatment: chemotherapy and other considerations. Brain Imaging Behav. (2013) 7:374–87. 10.1007/s11682-013-9256-x 23996156 PMC3869865

[24] Li M Caeyenberghs K. Longitudinal assessment of chemotherapy-induced changes in brain and cognitive functioning: a systematic review. Neurosci Biobehav Rev. (2018) 92:304–17. 10.1016/j.neubiorev.2018.05.019 29791867

[25] Ruiter MB de Reneman L Boogerd W Veltman DJ Caan M Douaud G . Late effects of high-dose adjuvant chemotherapy on white and gray matter in breast cancer survivors: converging results from multimodal magnetic resonance imaging. Hum Brain Mapp. (2012) 33:2971–83. 10.1002/hbm.21422 22095746 PMC6870296

[26] Peukert X Steindorf K Schagen SB Runz A Meyer P Zimmer P. Hippocampus-related cognitive and affective impairments in patients with breast cancer-a systematic review. Front Oncol. (2020) 10:147. 10.3389/fonc.2020.00147 32154164 PMC7046686

[27] Joisten N Kummerhoff F Koliamitra C Schenk A Walzik D Hardt L . Exercise and the Kynurenine pathway: Current state of knowledge and results from a randomized cross-over study comparing acute effects of endurance and resistance training. Exerc Immunol Rev. (2020) 26:24–42. 32139353

[28] Bruno A Dolcetti E Rizzo FR Fresegna D Musella A Gentile A . Inflammation-associated synaptic alterations as shared threads in depression and multiple sclerosis. Front Cell Neurosci. (2020) 14:169. 10.3389/fncel.2020.00169 32655374 PMC7324636

[29] Bedillion MF Ansell EB Thomas GA. Cancer treatment effects on cognition and depression: The moderating role of physical activity. Breast. (2019) 44:73–80. 10.1016/j.breast.2019.01.004 30685529

[30] Lange M Joly F Vardy J Ahles T Dubois M Tron L . Cancer-related cognitive impairment: an update on state of the art, detection, and management strategies in cancer survivors. Ann Oncol. (2019) 30:1925–40. 10.1093/annonc/mdz410 31617564 PMC8109411

[31] Park HS Kim CJ Kwak HB No MH Heo JW Kim TW. Physical exercise prevents cognitive impairment by enhancing hippocampal neuroplasticity and mitochondrial function in doxorubicin-induced chemobrain. Neuropharmacology. (2018) 133:451–61. 10.1016/j.neuropharm.2018.02.013 29477301

[32] Witlox L Schagen SB Ruiter MB de Geerlings MI Peeters PHM Koevoets EW . Effect of physical exercise on cognitive function and brain measures after chemotherapy in patients with breast cancer (PAM study): protocol of a randomised controlled trial. BMJ Open. (2019) 9:e028117. 10.1136/bmjopen-2018-028117 31227537 PMC6597001

[33] Hayes SC Newton RU Spence RR Galvão DA. The Exercise and Sports Science Australia position statement: Exercise medicine in cancer management. J Sci Med Sport. (2019) 22:1175–99. 10.1016/j.jsams.2019.05.003 31277921

[34] Zou LY Yang L He XL Sun M Xu JJ. Effects of aerobic exercise on cancer-related fatigue in breast cancer patients receiving chemotherapy: a meta-analysis. Tumour Biol. (2014) 35:5659–67. 10.1007/s13277-014-1749-8 24570186

[35] van Vulpen JK Peeters PHM Velthuis MJ van der Wall E May AM. Effects of physical exercise during adjuvant breast cancer treatment on physical and psychosocial dimensions of cancer-related fatigue: a meta-analysis. Maturitas. (2016) 85:104–11. 10.1016/j.maturitas.2015.12.007 26857888

[36] Furmaniak AC Menig M Markes MH. Exercise for women receiving adjuvant therapy for breast cancer. Cochrane Database Syst Rev. (2016) 9:CD005001. 10.1002/14651858.CD005001.pub3 27650122 PMC6457768

[37] Feng Y Tuluhong D Shi Z Zheng LJ Chen T Lu GM . Postchemotherapy hippocampal functional connectivity patterns in patients with breast cancer: a longitudinal resting state functional MR imaging study. Brain Imaging Behav. (2020) 14:1456–67. 10.1007/s11682-019-00067-x 30877468

[38] Apple AC Schroeder MP Ryals AJ Wagner LI Cella D Shih PA . Hippocampal functional connectivity is related to self-reported cognitive concerns in breast cancer patients undergoing adjuvant therapy. NeuroImage Clin. (2018) 20:110–8. 10.1016/j.nicl.2018.07.010 30094161 PMC6077172

[39] Cheng H Li W Gong L Xuan H Huang Z Zhao H . Altered resting-state hippocampal functional networks associated with chemotherapy-induced prospective memory impairment in breast cancer survivors. Sci Rep. (2017) 7:45135. 10.1038/srep45135 28327626 PMC5361087

[40] Zimmer P Schmidt ME Prentzell MT Berdel B Wiskemann J Kellner KH . Resistance exercise reduces kynurenine pathway metabolites in breast cancer patients undergoing radiotherapy. Front Oncol. (2019) 9:962. 10.3389/fonc.2019.00962 31612110 PMC6773833

[41] Prakash RS Voss MW Erickson KI Kramer AF. Physical activity and cognitive vitality. Annu Rev Psychol. (2015) 66:769–97. 10.1146/annurev-psych-010814-015249 25251492

[42] Erickson KI Hillman CH Kramer AF. Physical activity, brain, and cognition. Curr Opin Behav Sci. (2015) 4:27–32. 10.1016/j.cobeha.2015.01.005

[43] Stern Y MacKay-Brandt A Lee S McKinley P McIntyre K Razlighi Q . Effect of aerobic exercise on cognition in younger adults: a randomized clinical trial. Neurology. (2019) 92:e905–16. 10.1212/WNL.0000000000007003 30700591 PMC6404470

[44] Paillard T Rolland Y Souto Barreto P de. Protective Effects of Physical Exercise in Alzheimer's Disease and Parkinson's Disease: a narrative review. J Clin Neurol. (2015) 11:212–9. 10.3988/jcn.2015.11.3.212 26174783 PMC4507374

[45] Mattson MP. Lifelong brain health is a lifelong challenge: from evolutionary principles to empirical evidence. Ageing Res Rev. (2015) 20:37–45. 10.1016/j.arr.2014.12.011 25576651 PMC4346441

[46] Tari AR Norevik CS Scrimgeour NR Kobro-Flatmoen A Storm-Mathisen J Bergersen LH . Are the neuroprotective effects of exercise training systemically mediated? Prog Cardiovasc Dis. (2019) 62:94–101. 10.1016/j.pcad.2019.02.003 30802460

[47] Smith PJ Blumenthal JA Hoffman BM Cooper H Strauman TA Welsh-Bohmer K . Aerobic exercise and neurocognitive performance: a meta-analytic review of randomized controlled trials. Psychosom Med. (2010) 72:239–52. 10.1097/PSY.0b013e3181d14633 20223924 PMC2897704

[48] Apple AC Ryals AJ Alpert KI Wagner LI Shih PA Dokucu M . Subtle hippocampal deformities in breast cancer survivors with reduced episodic memory and self-reported cognitive concerns. Neuroimage Clin. (2017) 14:685–91. 10.1016/j.nicl.2017.03.004 28377882 PMC5369871

[49] Wang L Apple AC Schroeder MP Ryals AJ Voss JL Gitelman D . Reduced prefrontal activation during working and long-term memory tasks and impaired patient-reported cognition among cancer survivors postchemotherapy compared with healthy controls. Cancer. (2016) 122:258–68. 10.1002/cncr.29737 26484435 PMC4707984

[50] Voss MW Heo S Prakash RS Erickson KI Alves H Chaddock L . The influence of aerobic fitness on cerebral white matter integrity and cognitive function in older adults: results of a one-year exercise intervention. Hum Brain Mapp. (2013) 34:2972–85. 10.1002/hbm.22119 22674729 PMC4096122

[51] Fardell JE Vardy J Shah JD Johnston IN. Cognitive impairments caused by oxaliplatin and 5-fluorouracil chemotherapy are ameliorated by physical activity. Psychopharmacology (Berl). (2012) 220:183–93. 10.1007/s00213-011-2466-2 21894483

[52] Knaepen K Goekint M Heyman EM Meeusen R. Neuroplasticity - exercise-induced response of peripheral brain-derived neurotrophic factor: a systematic review of experimental studies in human subjects. Sports Med. (2010) 40:765–801. 10.2165/11534530-000000000-00000 20726622

[53] Cabral-Santos C Castrillón CIM Miranda RAT Monteiro PA Inoue DS Campos EZ . Inflammatory Cytokines and BDNF Response to High-Intensity Intermittent Exercise: Effect the Exercise Volume. Front Physiol. (2016) 7:509. 10.3389/fphys.2016.00509 27867360 PMC5095487

[54] Ramos JS Dalleck LC Tjonna AE Beetham KS Coombes JS. The impact of high-intensity interval training versus moderate-intensity continuous training on vascular function: a systematic review and meta-analysis. Sports Med. (2015) 45:679–92. 10.1007/s40279-015-0321-z 25771785

[55] Mijwel S Backman M Bolam KA Jervaeus A Sundberg CJ Margolin S . Adding high-intensity interval training to conventional training modalities: optimizing health-related outcomes during chemotherapy for breast cancer: the OptiTrain randomized controlled trial. Breast Cancer Res Treat. (2018) 168:79–93. 10.1007/s10549-017-4571-3 29139007 PMC5847033

[56] Oberste M Schaffrath N Schmidt K Bloch W Jäger E Steindorf K . Protocol for the “Chemobrain in Motion - study” (CIM - study): a randomized placebo-controlled trial of the impact of a high-intensity interval endurance training on cancer related cognitive impairments in women with breast cancer receiving first-line chemotherapy. BMC Cancer. (2018) 18:1071. 10.1186/s12885-018-4992-3 30400840 PMC6220507

[57] Gentry AL Erickson KI Sereika SM Casillo FE Crisafio ME Donahue PT . Protocol for Exercise Program in Cancer and Cognition (EPICC): A randomized controlled trial of the effects of aerobic exercise on cognitive function in postmenopausal women with breast cancer receiving aromatase inhibitor therapy. Contemp Clin Trials. (2018) 67:109–15. 10.1016/j.cct.2018.02.012 29501739 PMC5877817

[58] Gokal K. Walking protects against decline in self-reported cognitive functioning among breast cancer patients undergoing chemotherapy: a small randomised controlled trial: Open Science Framework. (2018). 10.1371/journal.pone.0206874 30485297 PMC6261560

[59] Bolam KA Mijwel S Rundqvist H Wengström Y. Two-year follow-up of the OptiTrain randomised controlled exercise trial. Breast Cancer Res Treat. (2019) 175:637–48. 10.1007/s10549-019-05204-0 30915663 PMC6534518

[60] Lee K Kang I Mack WJ Mortimer J Sattler F Salem G . Feasibility of high intensity interval training in patients with breast Cancer undergoing anthracycline chemotherapy: a randomized pilot trial. BMC Cancer. (2019) 19:653. 10.1186/s12885-019-5887-7 31269914 PMC6610838

[61] Beyer MK Bronnick KS Hwang KS Bergsland N Tysnes OB Larsen JP . Verbal memory is associated with structural hippocampal changes in newly diagnosed Parkinson's disease. J Neurol Neurosurg Psychiatry. (2013) 84:23–8. 10.1136/jnnp-2012-303054 23154124 PMC4041694

[62] Hoseth EZ Westlye LT Hope S Dieset I Aukrust P Melle I . Association between cytokine levels, verbal memory and hippocampus volume in psychotic disorders and healthy controls. Acta Psychiatr Scand. (2016) 133:53–62. 10.1111/acps.12467 26189721

[63] Wefel JS Schagen SB. Chemotherapy-related cognitive dysfunction. Curr Neurol Neurosci Rep. (2012) 12:267–75. 10.1007/s11910-012-0264-9 22453825

[64] Oberste M Bloch W Hübner ST Zimmer P. Do reported effects of acute aerobic exercise on subsequent higher cognitive performances remain if tested against an instructed self-myofascial release training control group? A randomized controlled trial. PLoS ONE. (2016) 11:e0167818. 10.1371/journal.pone.0167818 27930706 PMC5145178

[65] Song D Yu DSF. Effects of a moderate-intensity aerobic exercise programme on the cognitive function and quality of life of community-dwelling elderly people with mild cognitive impairment: a randomised controlled trial. Int J Nurs Stud. (2019) 93:97–105. 10.1016/j.ijnurstu.2019.02.019 30901716

[66] Travier N Velthuis MJ Steins Bisschop CN van den Buijs B Monninkhof EM Backx F . Effects of an 18-week exercise programme started early during breast cancer treatment: a randomised controlled trial. BMC Med. (2015) 13:121. 10.1186/s12916-015-0362-z 26050790 PMC4461906

[67] Sheill G Guinan E Brady L Hevey D Hussey J. Exercise interventions for patients with advanced cancer: a systematic review of recruitment, attrition, and exercise adherence rates. Palliat Support Care. (2019) 17:686–96. 10.1017/S1478951519000312 31109383

[68] Hoover JC Alenazi AM Alshehri MM Alqahtani BA Alothman S Sarmento C . Recruiting and retaining patients with breast cancer in exercise trials: a meta-analysis. Transl J ACSM. (2021) 6:413. 10.1249/TJX.0000000000000149

[69] Campbell KL Winters-Stone KM Wiskemann J May AM Schwartz AL Courneya KS . Exercise guidelines for cancer survivors: consensus statement from international multidisciplinary roundtable. Med Sci Sports Exerc. (2019) 51:2375–90. 10.1249/MSS.0000000000002116 31626055 PMC8576825

[70] Wonisch M Berent R Klicpera M Laimer H Marko C Schwann H . Praxisleitlinien Ergometrie. Journal für Kardiologie. (2008) 15:3–17.

[71] Tschakert G Hofmann P. High-intensity intermittent exercise: methodological and physiological aspects. Int J Sports Physiol Perform. (2013) 8:600–10. 10.1123/ijspp.8.6.600 23799827

[72] Andreotti C Root JC Schagen SB McDonald BC Saykin AJ Atkinson TM . Reliable change in neuropsychological assessment of breast cancer survivors. Psychooncology. (2016) 25:43–50. 10.1002/pon.3799 25808921 PMC4580503

