# Supplementary material for: Protocol for the Exercise, Cancer and Cognition – The ECCO-Study: A Randomized Controlled Trial of Simultaneous Exercise During Neo-/Adjuvant Chemotherapy in Breast Cancer Patients and Its Effects on Neurocognition
Source: Front Neurol. 2022 Mar 25;13:777808. doi: 10.3389/fneur.2022.777808 (PMC8990905; doi:10.3389/fneur.2022.777808)
Supplement: Supplementary file 1 [file Table_1.DOCX]

# Appendix: Assessment Table – Timeline (Months)

| **Variable** | **Baseline** | **Interim Assessments** | | | | | **End of Study Intervention** | **FU-Visits** | |
| --- | --- | --- | --- | --- | --- | --- | --- | --- | --- |
|  |  | **2** | **4** | **6** | **8** | **10** | **12** | **24** | **36** |
| Demographic data | X |  |  |  |  |  |  |  |  |
| Standing Height | X |  |  |  |  |  |  |  |  |
| Weight | X | X | X | X | X | X | X | X | X |
| BMI | X | X | X | X | X | X | X | X | X |
| ECOG | X | X | X | X | X | X | X | X | X |
| Pregnancy Test | X | X | X | X | X | X | X |  |  |
| Fatigue | X | X | X | X | X | X | X | X | X |
| Pulse/ Heart Rate | X | X | X | X | X | X | X | X | X |
| Blood Pressure | X |  |  | X |  |  | X |  |  |
| ECG | X | X | X | X | X | X | X | X | X |
| Echocardiography | X |  |  |  |  |  | X |  |  |
| Concomitant Diseases | X | X | X | X | X | X | X | X | X |
| Medication | X | X | X | X | X | X | X | X | X |
| Training Activity per Week (h) | X | X | X | X | X | X | X | X | X |
| MET/ Week | X | X | X | X | X | X | X | X | X |
| Training Days/ Week | X | X | X | X | X | X | X | X | X |
| CVLT (*PE) | X |  |  |  |  |  | X |  |  |
| Additional tests to differ in neurocognitive function as described in chapter (10.2) | X |  |  |  |  |  | X |  |  |
| Head MRI | X |  |  |  |  |  | X |  |  |
| BDI-II | X |  |  | X |  |  | X | X | X |
| BAI | X |  |  | X |  |  | X | X | X |
| Biomarker Analysis | X |  |  | X |  |  | X |  |  |
| PSQI | X |  |  | X |  |  | X | X | X |
| MFI-20 | X |  |  | X |  |  | X | X | X |
| FACT Cog | X |  |  | X |  |  | X | X | X |
| FACT F | X |  |  | X |  |  | X | X | X |
| EORTC QLQ | X |  |  | X |  |  | X | X | X |
| Progression |  | X | X | X | X | X | X | X | X |
| Side Effects/ Adverse Events |  | X | X | X | X | X | X | X | X |
| Spiroergometry | X |  |  | X |  |  | X |  |  |
| GPAQ | X |  |  | X |  |  | X | X | X |
| BIA | X |  |  | X |  |  | X |  |  |
| WtHR | X |  |  | X |  |  | X |  |  |

| **W 1-4** | **W 5-8** | **W 9-12** | **W 13-16** | **W 17-20** | **W 21-24** | **W 25-28** | **W 29-32** | **W 33-36** | **W 37-40** | **W 41-44** | **W 45-48** | **W 49-52** |
| --- | --- | --- | --- | --- | --- | --- | --- | --- | --- | --- | --- | --- |
| 1x Stability/Upper body strength (S/U) = 3MET | 1x S/U | 1x S/U | 1x S/U | 1x S/U | 1x S/U | 1x S/U | 1x S/U | 1x S/U | 1x S/U | 1x S/U | 1x S/U | 1x S/U |
| 1x Endurance Exercise (EEGA1) (>30min) = 5MET or 1 x Intervall Training (IT) - (10 intervalls according to 1 min >85%PMax.) = 5MET | 1x  EEGA1 (>45m) or 1 IT/ 13 I | 1x EEGA1 (>30m) | 1 x EEGA1 (>45m) | 1 x EEGA1 (>45m) | 1 x EEGA1 (>45m) | 2 x EEGA1 (>45m) | 2 x EEGA1 (>45m) | 2 x EEGA1 (>45m) | 2 x EEGA1 (>45m) | 2 x EEGA1 (>45m) | 2 x EEGA1 (>45m) | 2 x EEGA1 (>45m) |
|  |  | 1 IT á 10 | 1 IT á 13 | 1 IT á 13 | 1 IT á 13 | 1 IT á 15 | 1 IT á 15 | 1 IT á 15 | 1 IT á 15 | 1 IT á 15 | 1 IT á 15 | 1 IT á 15 |
| **8 MET** | **11 MET** | **13 MET** | **15 MET** | **15 MET** | **15 MET** | **22 MET** | **22 MET** | **22 MET** | **22 MET** | **22 MET** | **22 MET** | **22 MET** |
